# *Tremella fuciformis* polysaccharide reduces obesity in high-fat diet-fed mice by modulation of gut microbiota

**DOI:** 10.3389/fmicb.2022.1073350

**Published:** 2022-12-05

**Authors:** Gang He, Tangcong Chen, Lifen Huang, Yiyuan Zhang, Yanjiao Feng, Shaokui Qu, Xiaojing Yin, Li Liang, Jun Yan, Wei Liu

**Affiliations:** Key Laboratory of Medicinal and Edible Plants Resources Development of Sichuan Education Department, Sichuan Industrial Institute of Antibiotics, School of Pharmacy, Chengdu University, Chengdu, China

**Keywords:** *Tremella fuciformis* polysaccharide, obesity, gut microbiota, inflammation, SCFAs, microbe-gut-brain axis

## Abstract

Obesity is a metabolic disease associated with gut microbiota and low-grade chronic inflammation. *Tremella fuciformis* is a medicinal and edible fungus; polysaccharide (TP) is the main active component, which has a variety of biological activities, such as hypoglycemic and hypolipidemic. However, the anti-obesity effects and potential mechanisms of TP have never been reported. This study was conducted to elucidate the inhibitory effect of TP on high-fat diet (HFD)-induced obesity in mice. Mice were split into five groups: normal chow diet (*NCD*) group, *NCD_TP_H* group, *HFD* group, *HFD_TP_L* group and *HFD_TP_H* group. Our study showed that TP inhibited high-fat diet-induced weight gain and fat accumulation in mice and reduced blood glucose, hyperlipidemia and inflammation. TP also improved gut microbiota disorders by reducing the *Firmicutes*/*Bacteroidetes* ratio and modulating the relative abundance of specific gut microbiota. We also found that the anti-obesity and gut microbiota-modulating effects of TP could be transferred to HFD-fed mice *via* faecal microbiota transplantation (FMT), confirming that the gut microbiota was one of the targets of TP for obesity inhibition. Further studies showed that TP increased the production of short-chain fatty acids and the secretion of intestinal hormones. Our studies showed that TP inhibited obesity by modulating inflammation and the microbe-gut-brain axis, providing a rationale for developing TP to treat obesity and its complications.

## Introduction

Obesity is associated with many health problems and reduced life expectancy (Hoyt et al., 2014; Fan and Pedersen, 2021). Numerous studies have shown that obesity increases the risk of type 2 diabetes, nonalcoholic fatty liver disease, cardiovascular disease, and several types of cancer (Font-Burgada et al., 2016; Song et al., 2021). The high prevalence of obesity is a significant public health threat, and it is estimated that by 2030, more than 50% of the global population will be overweight or obese (Kelly et al., 2008). Obesity and the resulting chronic diseases significantly threaten people’s health and cause a heavy economic and medical burden. Therefore, treating and preventing obesity is a great challenge in modern society.

The gut microbiome represents the trillions of microbes in the gut, including thousands of species of bacteria (Sender et al., 2016; Hall et al., 2017; Fan and Pedersen, 2021). A large number of studies have shown that the gut microbiota is closely related to the development of obesity and related metabolic diseases (DiBaise et al., 2008; Ridaura et al., 2013; Sommer and Bckhed, 2013; Federico et al., 2017). It is mainly reflected in the following aspects: (1) changes in gut microbiota structure, especially the ratio of *Firmicutes* and *Bacteroidetes* (Turnbaugh et al., 2006; Goodman et al., 2011), and changes in the abundance of some harmful bacteria affect the development of obesity. (2) Disruption of the gut microbiota increases the amount of lipopolysaccharide (LPS) produced by bacteria, which disrupts the gut barrier (Cani et al., 2008; Kyung-Ah et al., 2012). Large amounts of LPS enter the bloodstream and induce metabolic inflammation (Cani et al., 2007), ultimately leading to metabolic syndromes such as obesity (Noriyuki et al., 2011; Amandine Everard and Cani, 2013). (3) The gut microbiota ferments and digests dietary fibre, polysaccharides, and other carbohydrates to produce large amounts of short-chain fatty acids (SCFAs; Fellow and Shanahan, 2014). SCFAs were absorbed by the gut and release more gut hormones, such as glucagon-like peptide-1 (GLP-1) and peptide YY (PYY), resulting in lower food intake (Freeland and Wolever, 2010; Lin et al., 2012), thereby curbing obesity (Keenan et al., 2015; Reiner et al., 2016; Namkoong et al., 2017).

*Tremella fuciformis*. Bark has been used in China for thousands of years due to its unique flavour and positive role in disease prevention and treatment (Wu et al., 2019; Yuan et al., 2022). As one of its active components, Tremella polysaccharide (TP) has many pharmacological effects, such as lowering blood glucose and lipid (Kim et al., 2009; Bach et al., 2015; Chiu et al., 2022; Zhang et al., 2023)and immunomodulation (Shi et al., 2014; Xiao et al., 2021; Khan et al., 2022; Xie et al., 2022). In addition, the anti-radiation (Xu et al., 2012), anti-ageing (Tao et al., 2017) and antioxidant (Chen, 2010) effects of TP have also been reported. Recent studies have reported that TP can reduce atopic dermatitis in mice by regulating gut microbiota (Xie et al., 2022); however, little research has been done on whether and how TP prevents and treats obesity.

Therefore, we examined whether TP could suppress high-fat diet-induced obesity in mice. Our results showed that TP suppressed obesity and improved glucose metabolism in mice. We speculated that the anti-obesity effect of TP is related to gut microbes, and our results showed that TP increases the diversity of gut microbes and improves gut microbial disorders. Moreover, the anti-obesity effect of TP could be transferred to HFD-fed mice *via* faecal microbiota transplantation, which further validated our hypothesis. By determining the content of short-chain fatty acids in faeces by GC–MS and the expression of associated genes and proteins, we found that TP exerts its weight loss effects *via* the microbe-gut-brain axis.

## Materials and methods

### Materials and reagents

Feed formulas for the experimental mice included the normal control diet (containing 23.07% of protein,11.85% of fat and 65.08% carbohydrates,3.40 kcal/g), purchased from Beijing Keao Xieli Feed Co., Ltd., Beijing, China, and the high-fat diet (containing 23.25% of protein, 34.55% of fat and 27.20% of carbohydrates, 5.13 kcal/g), purchased from Jiangsu Pharmaceutical & Bioengineering Co., Ltd., Jiangsu, China. SPF-grade poplar wood shavings as mouse bedding purchased from Beijing Keao Xieli Feed Co., Ltd., Beijing, China. All other chemical reagents are at least of analytical grade.

### *Tremella fuciformis* polysaccharides prepared

*Tremella fuciformis* was collected from Sichuan Yudeyuan Ecological Agriculture Technology Co.Ltd., TongJiang, Sichuan, China. The fruit bodies were defatted with anhydrous ethanol and extracted water-soluble polysaccharides with 100 times pure water at 100°C for 2 h, Repeated the extraction twice; the mixed extract was filtered, concentrated and centrifuged to obtain the supernatant. Four times the amount of anhydrous ethanol was added to the supernatant, the polysaccharide was precipitated overnight and centrifuged to obtain TP crude polysaccharide, and the impurities were washed with anhydrous ethanol. Evaporate the ethanol, re-dissolve in pure water and use the TCA method to remove the protein. Dialysis (retention capacity of 8,000 Da) for 24 h, vacuum lyophilisation to obtain TP (Wu et al., 2019; Yuan et al., 2022). The phenol-sulphuric acid method determined the total sugar content using mannose as a standard (Wang et al., 2017).

### Characterised of *Tremella fuciformis* polysaccharide

Impurities in TP were analysed by the UV-2700i system of the Shimadzu UV–Vis spectrophotometer, and the wavelength was from 200 to 700 nm. The functional groups and glycosidic bonds of TP were determined by the Fourier transform-infrared (FT-IR) spectra (Thermo, American) with a resolution of 4 cm^−1^ in the 4,000–400 cm^−1^ region. TP (~0.5 mg) was mixed with KBr and pressed into thin slices with abrasive tools at room temperature (Yukun et al., 2018).

HPLC determined monosaccharide composition (Shu et al., 2019). In brief, the TP (typically 2 mg) was hydrolysed with 0.5 M sulphuric acid at 120°C under nitrogen for 4 h. The TP hydrolysate was neutralised with sodium hydroxide and then derivatised with 450 μl 1-phenyl-3-methyl-5-pyrazolone (PMP) solution (0.5 M, in methanol) and 450 μl of 0.3 M NaOH at 70°C for 30 min. The reaction was stopped by neutralisation with 450 μl of 0.3 M HCl and extraction with Chloroform. HPLC analyses were performed on a Diamonsil C18 (5 μm, 4.6 mm × 150 mm) at 25°C with detection at UV 245 nm. The mobile phase was aqueous 0.1 M potassium dihydrogen phosphate (solvent A) and acetonitrile (solvent B). A gradient of B from 45% to 100% in 40 min was used, and the injection volume was 10 μl. The standard monosaccharides (Man, Rha, GlcA, GalA, Glc, Gal, Xyl) were weighed to prepare a series of concentration solutions. The derivatization of monosaccharides and the chromatographic analysis were carried out according to the above method. The molar ratio of each monosaccharide in TP was calculated by the standard external method.

The molecular weight of the TP was confirmed by liquid chromatography on a Hitachi L-2000 system (Hitachi, Japan) with a YMC PACK-Diol 200 Å (300 mm × 8.0 mm, ID 5 μm,20 nm)column by elution with ultrapure water, column temperature 30°C, the flow rate of 0.8 ml/min, and the injection volume was 20 μl and detection by the refractive index (Meng et al., 2016).

### Animals and experimental design

Fifty male C57BL/6J mice (8-week-old, 20 ± 2 g) were purchased from SiPeiFu Biotechnology Co., Ltd., Beijing, China. Mice were housed at constant temperature and humidity (22 ± 2°C; 55 ± 5% humidity), with free access to food and water and a 12-h light/dark cycle. All animal procedures followed the Guidelines for Care and Use of Laboratory Animals of the National Institutes of Health. The Ethical Committee approved all animal experiments for the Protection of Laboratory Animals, Sichuan Industrial Institute of Antibiotics, Chengdu University (Chengdu, China; Approval Number: SIIA 20210702). After a week of acclimatisation, the mice were randomly divided into five groups (*n* = 10 per group): (1) *NCD* group (fed normal chow and gavaged with 0.9% saline daily); (2) *NCD_TP_H* group (fed normal chow and gavaged with 200 mg/kg of TP daily); (3) *HFD* group (fed high-fat chow and gavaged with 0.9% saline daily); (4) *HFD_TP_L* group (fed high-fat feed and gavage of 100 mg/kg of TP daily); (5) *HFD_TP_H* group (fed high-fat feed and gavage of 200 mg/kg of TP daily). The experiment lasted for 12 weeks, and the body weight and food intake of each mouse were recorded weekly. In the 12th week, all mice were fasted overnight, anaesthetized with sodium pentobarbital and then subjected to cervical dislocation for execution. Serum was collected, and tissues such as epididymal fat, perirenal fat, liver and brain were excised, weighed, and tissues were fixed in 4% formaldehyde, whilst the rest were rapidly frozen in liquid nitrogen for further analysis.

### Faecal microbiota transplantation

Eight-week-old male C57BL/6J mice were randomly divided into four groups (5 mice in each group) after acclimatisation, and all were fed with high-fat chow (Chang et al., 2015; Zhang et al., 2020). The faecal samples of the *NCD* group, *NCD_TP_H* group, *HFD* group and *HFD_TP_H* mice were collected. 100 mg of faecal samples were taken in 1 ml of 0.9% sterile saline, vortexed and mixed for 10 s, then centrifuged at 800*g* for 3 min, and the supernatant was collected as the grafted sample. Fresh samples were prepared within 10 min before gavage, and fresh samples (100 μl each) were gavaged daily for 2 months before mice were executed, and serum and fat were collected for the following step analysis.

### Biochemical analysis

The levels of TC, TG, LDL-C, and HDL-C in serum were measured according to the instructions of the kit (Jiancheng, Inc., Nanjing, China), and the levels of GLP-1, GLP-2, IL-6, LPS, and TNF-α in serum were measured with the Elisa kit (Ruikesi Biotechnology Co., Ltd., Chengdu, China).

### Oral glucose tolerance test

In the 11th week of the experiment, mice were fasted overnight (12 h; Zhang et al., 2020). A glucose solution of 1 g/kg body weight was gavaged, and blood glucose was measured after 0, 15, 30, 60, 90, and 120 min.

### RNA extraction and qRT-PCR analysis

Total RNA was extracted from the tissue using a Trizol reagent according to the kit instructions. Equal amounts of total RNA were used to synthesise cDNA with the EX RT kit (gDNA remover; Zoman Biotechnology Co., Ltd., Beijing, China). cDNA synthesised was evaluated using a real-time quantitative PCR system (StepOnePlus™ Real-Time PCR Detection System, Applied Biosystems, United States). The PCR was performed in duplicate at 95°C for 3 min and subjected to 40 cycles of 95°C for 120 s, 95°C for 5 s, and 60°C for 10 s. GAPDH was used as the reference gene. Data were analysed using the 2^−ΔΔCt^ method. The primers for the genes targeted in this study are listed in the Supplementary material (Supplementary Table 1).

### Western blotting

Total protein was obtained by lysing the tissue in ice-cold RIPA buffer containing phosphatase and protease inhibitors. The protein concentration was determined using the BCA protein assay kit, diluting each group of samples to the same protein concentration. Total protein was separated on a 12% SDS-PAGE gel and then transferred to polyvinylidene difluoride membranes (Immobilon TM-P; Millipore, United States). After being blocked with 5% non-fat milk in TBST for 1 h, the membrane was probed with primary antibody overnight at 4°C. The membranes were then probed with secondary antibodies for 1 h at room temperature and exposed using an ECL chemical enhancer. Quantitative analysis of optical density was performed using ImageJ software.

### Histopathological analysis

Mice epididymal fat was fixed overnight in 4% formaldehyde solution, dehydrated, embedded and sectioned, then stained with haematoxylin and eosin, and the sections were imaged using a digital trinocular camera (BA210 Digital, Motic Group Co., Ltd.) microscope system (Chen C. et al., 2018).

### Gas chromatography–mass spectrometry analysis

It quantified short-chain fatty acids in faeces using a Perkin Elmer gas chromatograph and mass spectrometer (Perkin Elmer Technologies, United States). Short-chain fatty acids were measured in faeces concerning the previously described method (Zhang et al., 2020). Briefly, weigh 200 mg of faeces accurately, add 0.5 ml of 25% methanol solution, homogenise and vortex vigorously for 5 min; centrifuge at 4°C for 20 min at 10,000 rpm; remove the supernatant and centrifuge (10,000*g*, 10 min) at 4°C; add 10 times the volume of 0.04 M HCl solution to the supernatant and leave overnight at 4°C in an airtight container. The samples were passed through a 0.22 μm filter membrane into a clean micro gas-phase vial. The samples were filtered using a PerkinElmer Gas Chromatography-Mass Spectrometer (Shelton, Connecticut 0648 United States) with a polar DB-FFAP capillary column (30 m × 0.25 mm i.d., a film thickness of 0.25 μm) before analysis. The carrier gas was high-purity helium (99.999%) at a flow rate of 1 ml/min, and the auxiliary carrier gas was high-purity hydrogen (99.999%). The detector temperature and inlet temperature were both 250°C. The initial temperature is 100°C, ramped up to 180°C at a rate of 5°C per minute and held for 2 min, then ramped up to 250°C at a rate of 5°C per minute and held for 5 min, and the shunt ratio is 10:1.

### Gut microbiota analysis

Bacterial DNA was isolated from the faecal using a MagPure Soil DNA LQ Kit (Magen, Guangdong, China) following the manufacturer’s instructions. DNA concentration and integrity were measured using a NanoDrop 2000 spectrophotometer (Thermo Fisher Scientific, Waltham, MA, United States) and agarose gel electrophoresis. PCR amplification of the V3-V4 hypervariable regions of the bacterial 16S rRNA gene was carried out in a 25 μl reaction using universal primer pairs (343F: 5′-TACGGRAGGCAGCAG-3′; 798R: 5′-AGGGTATCTAATCCT-3′). The PCR products were purified with Agencourt AMPure XP beads (Beckman Coulter Co., United States) and quantified using a Qubit dsDNA assay kit. The concentrations were then adjusted for sequencing. Sequencing was performed on an Illumina NovaSeq6000 with two paired-end read cycles of 250 bases each (Illumina Inc., San Diego, CA; OE Biotech Company; Shanghai, China). Raw sequencing data were in FASTQ format. Paired-end reads were then preprocessed using cutadapt software to detect and cut off the adapter. After trimming, paired-end reads were filtered low quality sequences, denoised, merged and detect and cut off the chimaera reads using DADA2 (Chao and Bunge, 2002) with the default parameters of QIIME2 (Hill et al., 2003). At last, the software output the representative reads and the ASV abundance table. The representative read of each ASV was selected using QIIME 2 package. All representative reads were annotated and blasted against Silva database Version 138 (or Unite; 16 s/18 s/ITS rDNA) using q2-feature-classifier with the default parameters. The microbial diversity in faecal samples was estimated using the alpha diversity that includes the Shannon index and Simpson index. The Unifrac distance matrix performed by QIIME2 software was used for unweighted Unifrac Principal coordinates analysis (PCoA) and phylogenetic tree construction. The 16S rRNA gene amplicon sequencing and analysis were conducted by OE Biotech Co., Ltd. (Shanghai, China).

### Statistical analysis

One-way analysis of variance and Tukey’s test were used to determine significant differences. All data were presented as the mean ± standard deviation, and a *p*-value <0.05 was considered statistically significant. Statistical analysis was performed using GraphPad Prism version 9.0 (San Diego, California, United States).

## Results

### Physicochemical properties of *Tremella fuciformis* polysaccharide

**Figure 1:**
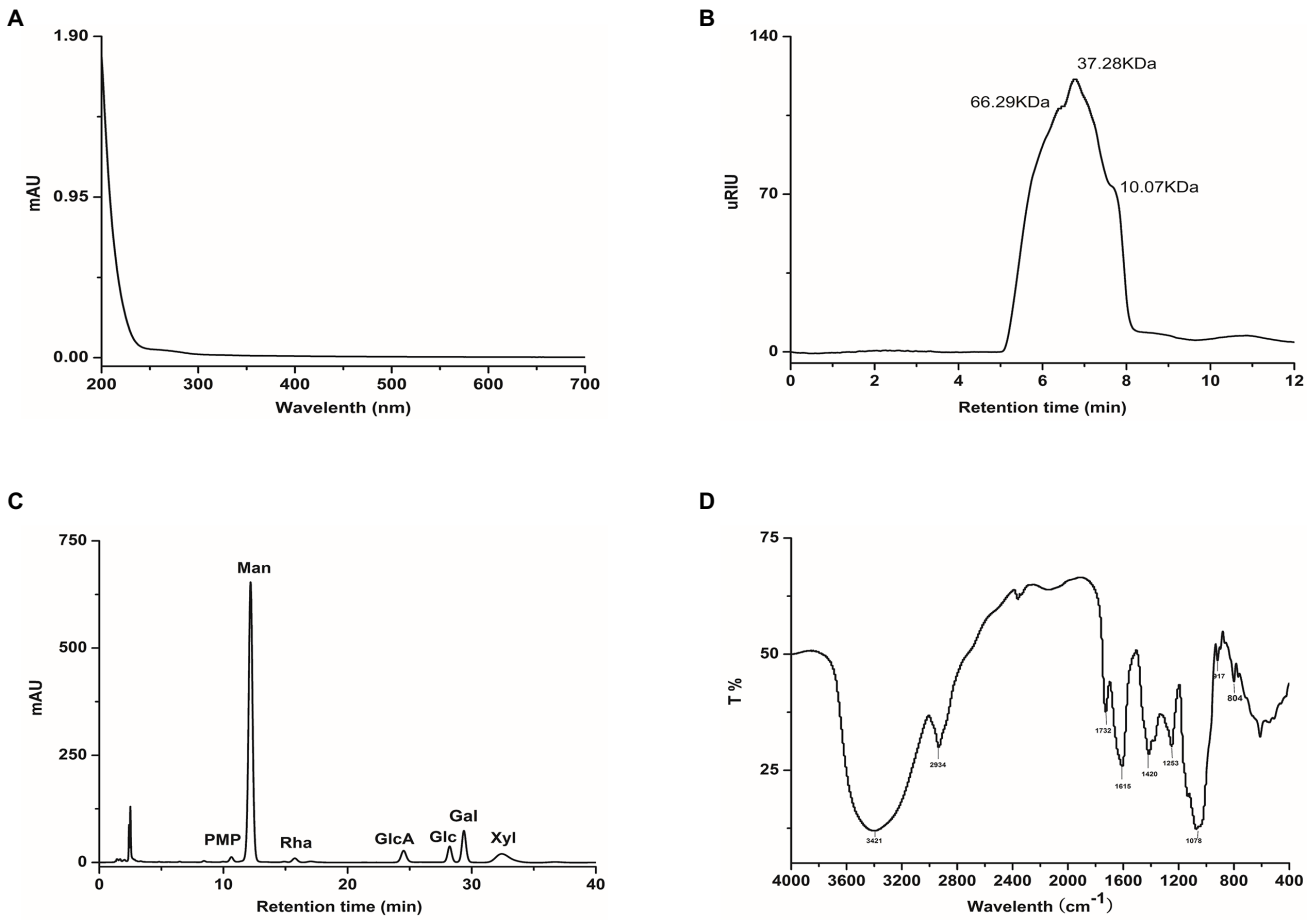
Characterise of TP, **(A)** UV–VIS absorption spectra, **(B)** High-performance size exclusion chromatograms, **(C)** HPLC analysis of monosaccharide composition, **(D)** FT-IR spectra.

The yield of TP from *Tremella fuciformis* fruit body by hot water extraction was about 9.8%. There were no prominent absorption peaks at 254 nm and 280 nm, indicating that the impurities in TP were entirely removed and TP had been refined (Figure 1A). The molecular weight distribution of TP ranges from 10.07 to 66.29 kDa, and the main molecular weight was 37.28 kDa (Figure 1B).TP was composed of Man, Rha, GlcA, Glc, Gal, and Xyl in a molar ratio of 15.95:0.31:2.47:1.20:3.18:1.72 (Figure 1C). The infrared spectrum of TP belongs to typical polysaccharide characteristics (Figure 1D); the absorption peaks around 3,421 and 2,934 cm^−1^ represent the stretching vibration of O-H and C-H, respectively. The absorption peaks around 1,732 and 1,615 cm^−1^ were the stretching vibrations of a carboxyl group (COO-), and the absorption peak around 1,078 cm^−1^ was owing to the stretching vibration of C-O-C and C-O-H bending vibration of pyranose ring (Wu et al., 2022; Yuan et al., 2022). The results showed that TP was a mannose-based heterosaccharide, and its monosaccharide composition was similar to the previously reported Tremella polysaccharide, but the composition ratio was inconsistent (Wu et al., 2019; Gao et al., 2022).

### TP inhibits high-fat diet-induced obesity in mice

**Figure 2:**
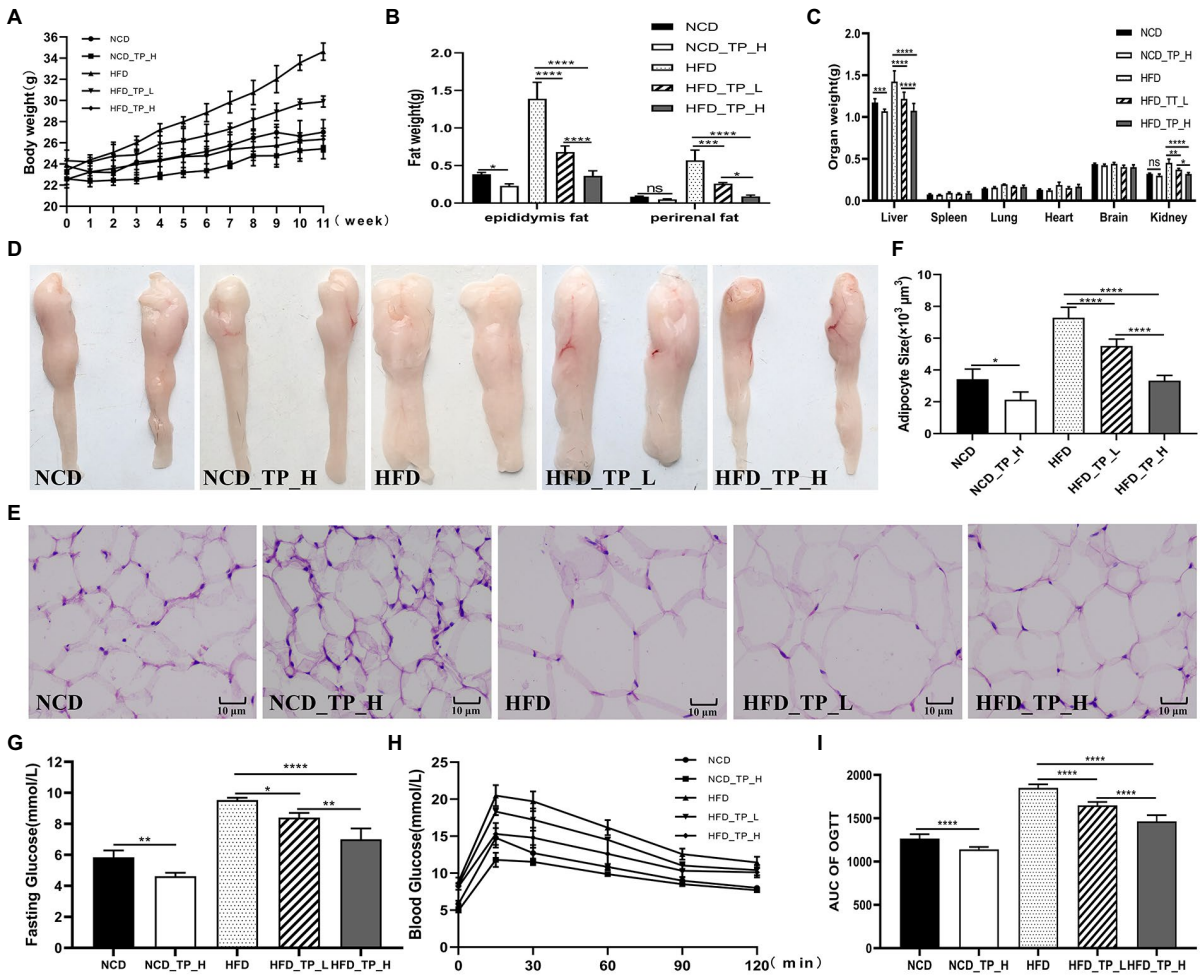
TP attenuated fat accumulation and hyperlipidemia in HFD-fed mice. **(A)** Body weight gain. **(B)** Epididymal fat and perirenal fat weight. **(C)** Weight of different organs. **(D)** Morphological observations of the epididymis fat. **(E)** Haematoxylin and eosin staining of epididymis fat. **(F)** Epididymal adipocyte size. **(G)** Fasting glucose. **(H)** Oral glucose tolerance test. **(I)** AUC of OGTT. Data were shown as means ± SD (*n* = 6). Statistical analysis was performed using one-way ANOVA; ns for *p* > 0.05; *p < 0.05; **p < 0.01; ***p < 0.001; and ****P < 0.0001.

After 12 weeks of feeding, we observed that feeding a high-fat diet caused a significant increase in body weight, fat weight, and liver weight in the mice, which was reversed with the TP intervention. In HFD-fed mice, TP inhibited weight gain and reduced fat accumulation in a dose-dependent manner (Figures 2A–C). Morphological observations showed that TP significantly inhibited high-fat diet-induced fat accumulation (Figure 2D) and adipocyte expansion (Figures 2E–F) in mice. These results revealed that TP reduced weight gain and fat accumulation in HFD-fed mice. TP ameliorated obesity in association with the modulation of lipid metabolism.

### TP improved blood glucose and lipid metabolism in HFD-fed mice

At week 11 of the experiment, a glucose tolerance test was performed, and glucose tolerance was quantified as the area under the curve (AUC) from 0 to 120 min (Figures 2H–I); glucose levels at 15–120 min were higher in HFD-fed mice than in NCD-fed mice. Mice in the *HFD_TP_L* and *HFD_TP_H* groups had smaller glucose AUC and showed better glucose regulation compared to the *HFD* group. Table 1 showed the effect of TP on blood biochemical parameters in mice. We observed that HFD significantly increased fasting blood glucose (Figure 2G) and serum TG, TC, and LDL-C levels in mice, a trend that was reversed by feeding TP. Similarly, the feeding of TP significantly increased the serum levels of HDL-C, GLP-1, and GLP-2 compared to the *HFD* group, indicating a better regulation of blood glucose and lipids by TP.

**Table 1:** Effects of TP on metabolic syndrome in HFD-fed mice.

Parameters	NCD	NCD_TP_H	HFD	HFD_TP_L	HFD_TP_H
TC (mmol/L)	3.22 ± 0.16^d^	2.60 ± 0.23^e^	4.77 ± 0.14^a^	4.39 ± 0.19^b^	4.07 ± 0.14^c^
TG (mmol/L)	0.98 ± 0.09^c^	0.89 ± 0.02^d^	1.43 ± 0.03^a^	1.37 ± 0.03^b^	0.93 ± 0.03^d^
LDL-C (mmol/L)	1.34 ± 0.14^b^	0.35 ± 0.01^d^	2.02 ± 0.26^a^	0.83 ± 0.02^c^	0.45 ± 0.03^d^
HDL-C (mmol/L)	3.51 ± 0.79^b^	5.47 ± 0.74^a^	1.88 ± 0.12^c^	2.86 ± 0.22^b^	3.05 ± 0.39^b^
GLP-1 (pmol/L)	3.78 ± 0.24^ab^	4.22 ± 0.40^a^	3.15 ± 0.21^c^	3.61 ± 0.44^b^	4.06 ± 0.39^ab^
GLP-2 (pg/L)	982.21 ± 14.61^b^	1029.33 ± 2.70^a^	756.25 ± 13.57^d^	854.80 ± 28.13^c^	961.83 ± 33.18^b^
LPS (EU/L)	6.12 ± 0.11^b^	4.30 ± 0.12^d^	6.82 ± 0.18^a^	5.47 ± 0.26^c^	5.33 ± 0.12^c^
TNF-α (pg/mL)	130.79 ± 1.31^b^	109.81 ± 1.68^e^	138.39 ± 1.50^a^	127.50 ± 0.62^c^	125.54 ± 0.14^d^
IL-6 (pg/mL)	29.71 ± 0.19^b^	22.60 ± 0.13^d^	45.55 ± 0.60^a^	29.97 ± 0.57^b^	27.48 ± 0.61^c^

NCD, normal chow diet; NCD_TP_H, normal chow diet with 200 mg/kg of TP; HFD, high fat diet; HFD_TP_L, high fat diet with 100 mg/kg of TP; HFD_TP_H, high fat diet with 200 mg/kg of TP. Data were expressed as mean ± SD (*n* = 6). Significant differences were evaluated by one-way analysis of variance (ANOVA). Comparison of each parameter between the five groups, with different superscript letters indicating significant differences (*p* < 0.05).

### TP reversed HFD-induced gut microbiota dysbiosis

**Figure 3:**
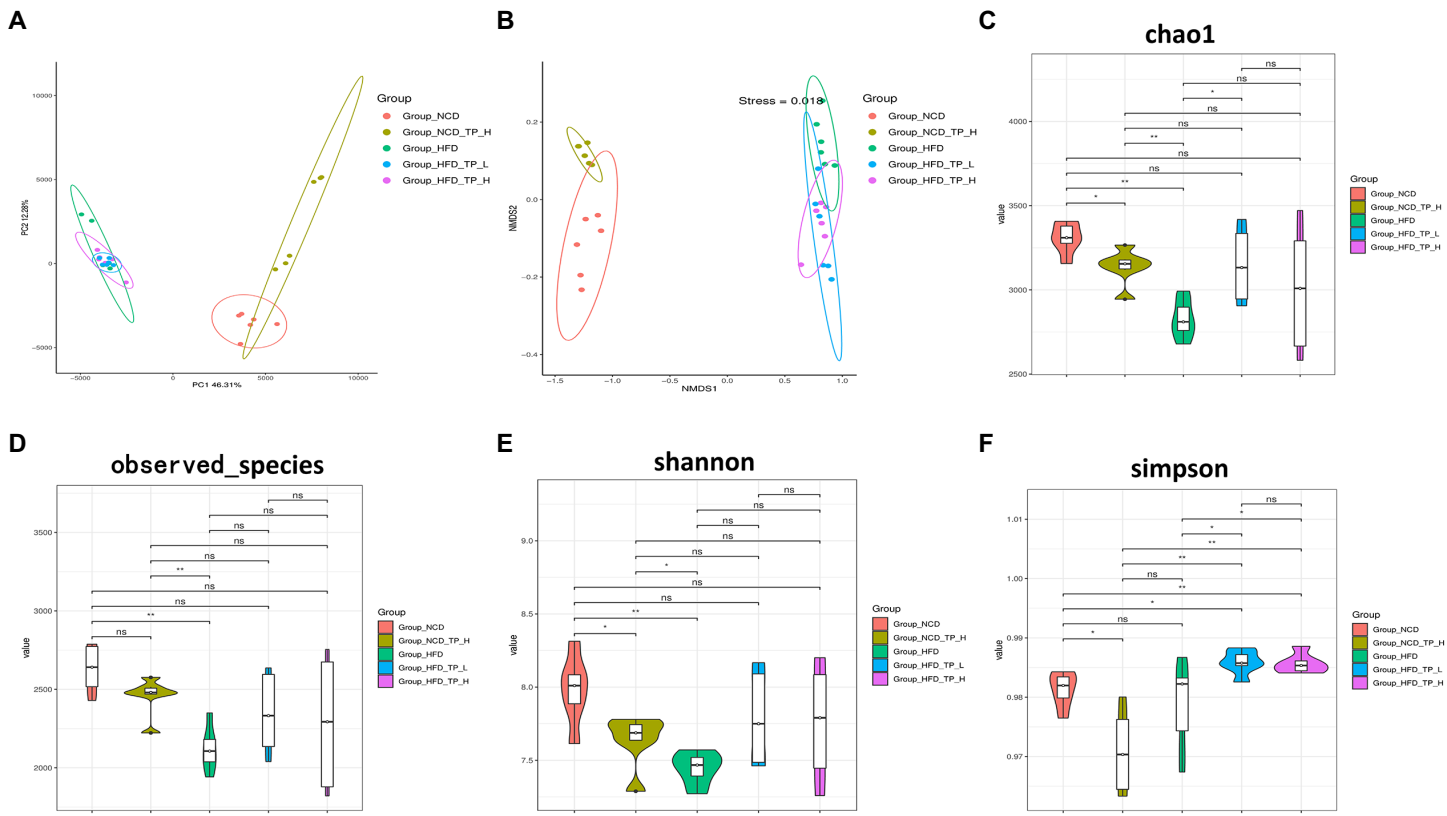
TP modulated the diversity of the gut microbiota. Beta diversity of faecal microbiota of the five groups, including **(A)** principal components analysis (PCoA) plot and non-metric multidimensional scaling (NMDS) analysis. **(B)** Alpha Diversity-related boxplot analysis, including **(C)** chao1, **(D)** observed species, **(E)** shannon and **(F)** simpson index. Data were shown as means ± SD (*n* = 6). Statistical analysis was performed using one-way ANOVA; ns for *p* > 0.05; *p < 0.05; **p < 0.01; ***p < 0.001; and ****p < 0.0001.

Gut microbiota plays a causal role in the development of metabolic syndrome caused by obesity and obesity-related metabolic disorders, and its diversity and changes are one of the important biological characteristics of the development of obesity (Federico et al., 2017; Fan and Pedersen, 2021). Therefore, we analysed the effect of TP on the composition of the gut microbiota by pyrophosphate sequencing-based analysis of bacterial 16S rRNA gene (regions V3-V4) in mice faeces. PCoA analysis showed an apparent clustering of the microbiota composition in each group (Figure 3A), with PCoA1 showing a 46.31% difference, mainly reflecting the effect of a normal and high-fat diet on changes in the structure of the gut microbiota; the vertical coordinate PCoA2 showed a 12.28% difference, which mainly reflects the effect of TP on the gut microbiota. NMDS plots showed that the high-fat diet significantly alters the structure of gut microbes in mice (Figure 3B). Alpha diversity analysis showed that the *HFD* group had the smallest chao1 values (Figure 3C) and obserced_species (Figure 3D) compared to the other four groups, and these two values were significantly higher after feeding TP. As shown in Figures 3E,F, the shannon and simpson indices were significantly higher after the TP intervention. These results suggested that the HFD reduced species’ diversity in mice’s gut microbiota. In contrast, feeding TP significantly improved the gut microbiota, mainly in increased diversity.

**Figure 4:**
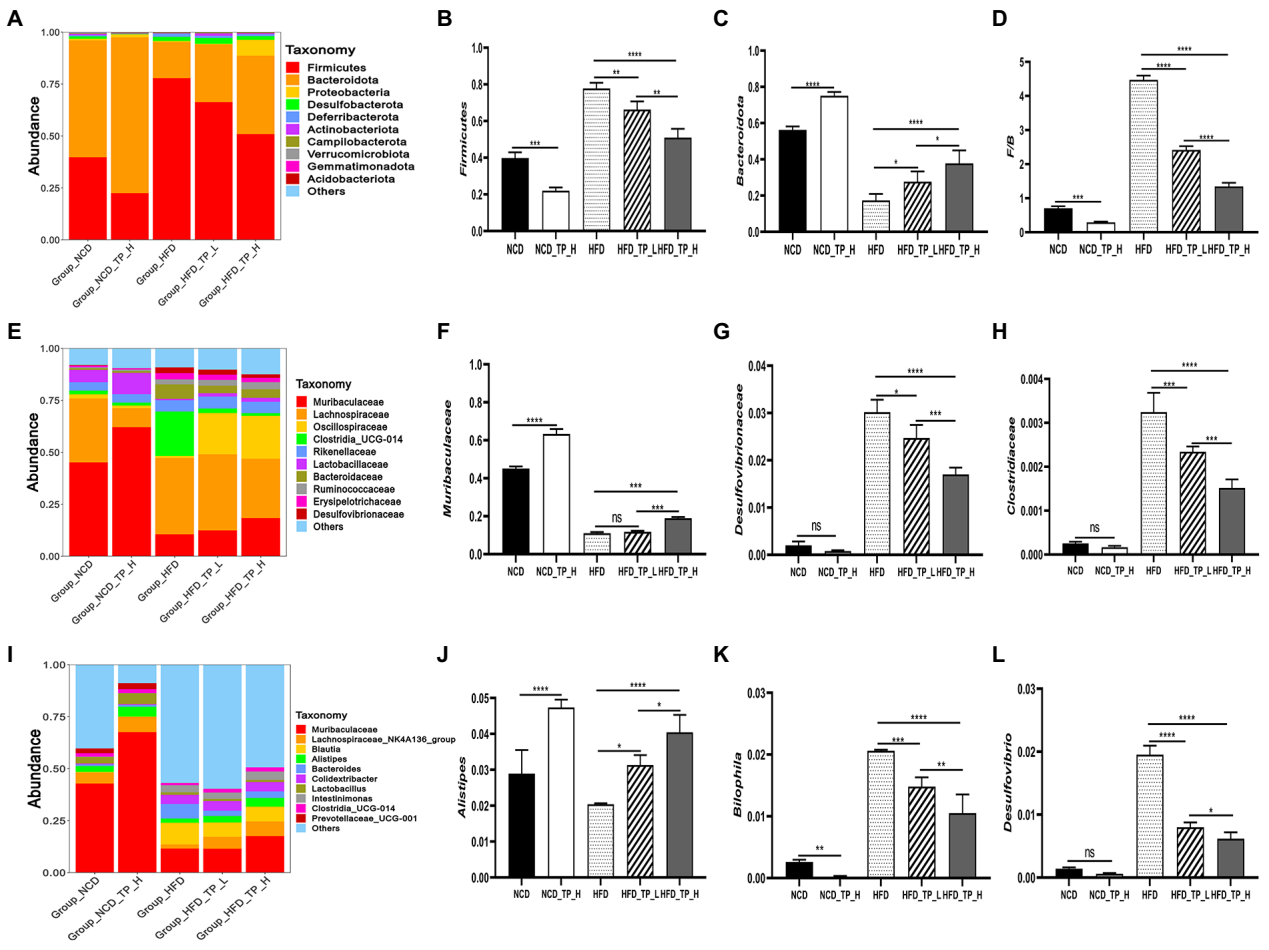
TP modulated the composition of the gut microbiota. **(A)** Phylum-, **(E)** family-, and **(I)** genus-level distribution of faecal microbiota. **(B,C)** Relative abundance of the phyla *Bacteroidota* and *Firmicutes*; **(D)** the ratio between the relative abundance of *Firmicutes* and *Bacteroidota*. **(F-H,J-L)** relative abundance of identified differential abundant bacterial groups at family- and genus-level. Data were shown as means ± SD (*n* = 6). Statistical analysis was performed using one-way ANOVA; ns for *p* > 0.05; *p < 0.05; **p < 0.01; ***p < 0.001; and ****p < 0.0001.

Furthermore, to investigate the regulatory effects of TP on the gut microbiota, we compared the relative abundance of the major taxa in the faecal microbiota of mice from five dietary groups. The gut microbiota composition has significant differences at phylum, family, and genus levels. At the phylum level, the histogram of the relative abundance of gut microbiome showed (Figure 4A) that all experimental groups included *Firmicutes, Bacteroidota, Desulfobacterota, Deferribacterota*, and *Proteobacteria*, with *Firmicutes* and *Bacteroidota* being the major phylum, accounting for over 80% of the total. Compared to the *NCD* group, The *HFD* group significantly altered the gut microbiota composition, characterised by a significant increase in the relative abundance of *Firmicutes* (Figure 4B) and a significant decrease in *Bacteroidota* (Figure 4C), resulting in a significant increase in the *Firmicutes*/*Bacteroidota* ratio. Interestingly, the *F*/*B* ratio also significantly decreased in the *NCD_TP_H* group compared to the *NCD* group (*p* < 0.05), and the *Firmicutes*/*Bacteroidota* ratios were significantly (*p* < 0.05) reduced in the *HFD_TP_L* and *HFD_TP_H* groups compared with the *HFD* group (Figure 4D) in a dose-dependent manner and tended to restore to the level of the *NCD* group.

At the family level, the most abundant microorganisms were mainly *Muribaculaceae*, *Lachnospiraceae*, *Oscillospiraceae*, *Rikenellaceae*, *Clostridia_UCG-014*, *Prevotellaceae*, *Erysipelotrichaceae*, *Ruminococcaceae*, and the high-fat diet significantly altered the relative abundance of the gut microbiota (Figure 4E). Compared with the *NCD* and TP intervention groups, the relative abundance of *Desulfovibrionaceae* (Figure 4G)*, Clostridia_UCG-014*, *Clostridiaceae* (Figure 4H), *Lachnospiraceae* and *Bacteroidaceae* was higher in the *HFD* group. Whilst *Muribaculaceae* (Figure 4F), *Oscillospiraceae*, *Lactobacillaceae* and *Prevotellaceae* had lower relative abundance. The abundance of gut microbiota in HFD and TP treatment groups was significantly altered at the genus level (Figure 4I). Compared with the *NCD* group and the TP intervention group, the abundance of beneficial microbiota was lower in the *HFD* group, such as *Muribaculaceae*, *Lachnospiraceae_NK4A136_group*, *Alistipes* (Figure 4J), *Colidextribacter*, *Lactobacillus*, *GCA-900066575*. Whilst the relative abundance of *Desulfovibrio* (Figure 4L), *Bilophila* (Figure 4K), *Blautia*, and *Intestinimonas* was higher in the *HFD* group. These results suggested that a high-fat diet altered the gut microbiota in mice, and TP intervention reversed the HFD-induced gut microbiota alterations, especially obesity-related microbes such as *Muribaculaceae*, *Lachnospiraceae*, *Alistipes*, *Bilophila*, and *Desulfovibrio*.

### Effect of TP on the SCFAs

The effect of TP on the content of SCFAs and BCFAs in the faeces of mice was shown in Table 2. The content of SCFAs in the faeces of mice in the *HFD* group was significantly lower than in the other four groups. In contrast, the levels of acetic, propionic and butyric acids were significantly higher after feeding TP (*p* < 0.05), There was no significant change in the content of valeric acid. In addition, the TP intervention also influenced the changes in the content of BCFAs, mainly in the form of a significant increase in the content of isobutyric acid (*p* < 0.05), but no significant change in the content of isovaleric acid.

**Table 2:** Faecal SCFAs contents of mice (μg/mg).

SCFAs	NCD	NCD_TP_H	HFD	HFD_TP_L	HFD_TP_H
Acetic acid	0.92 ± 0.01^b^	0.98 ± 0.02^a^	0.49 ± 0.03^d^	0.59 ± 0.01^c^	0.61 ± 0.02^c^
Propionic acid	0.42 ± 0.02^b^	0.55 ± 0.02^a^	0.09 ± 0.02^d^	0.11 ± 0.01^d^	0.17 ± 0.04^c^
Isobutyric acid	0.09 ± 0.01^b^	0.13 ± 0.01^a^	0.07 ± 0.01^c^	0.08 ± 0.01^bc^	0.12 ± 0.01^a^
Butyric acid	0.22 ± 0.01^b^	0.25 ± 0.01^a^	0.05 ± 0.01^e^	0.08 ± 0.01^d^	0.11 ± 0.01^c^
Isovaleric acid	0.08 ± 0.01^a^	0.07 ± 0.01^a^	0.07 ± 0.01^a^	0.08 ± 0.01^a^	0.05 ± 0.01^b^
Valeric acid	0.02 ± 0.01^a^	0.02 ± 0.01^a^	0.03 ± 0.01^a^	0.03 ± 0.00^a^	0.02 ± 0.00^a^

Data were expressed as mean ± SD (*n* = 6). Significant differences were evaluated by one-way analysis of variance (ANOVA). Comparison of each parameter between the five groups, with different superscript letters indicating significant differences (*p* < 0.05).

### TP regulated the expression of related genes and proteins

**Figure 5:**
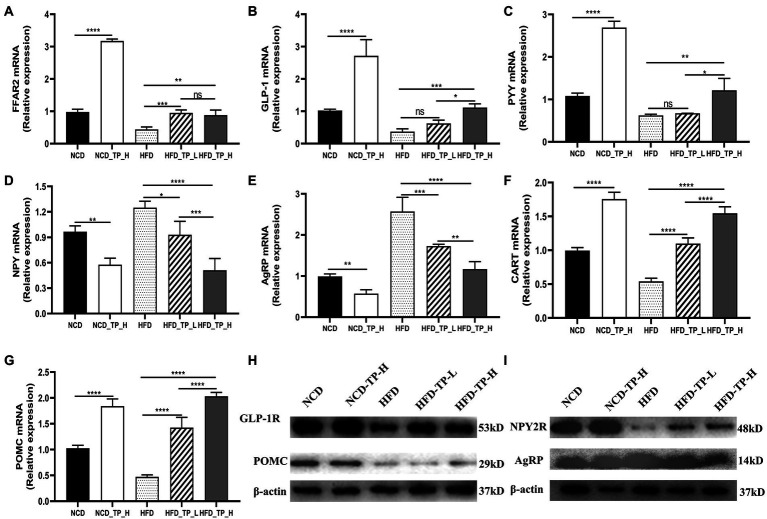
TP regulated the expression of related genes and proteins. Relative expression of **(A)** FFAR2, **(B)** GLP-1, and **(C)** PYY in the colon compared to the *NCD* group; relative expression of **(D)** NPY, **(E)** AGRP, **(F)** CART, and **(G)** POMC in the hypothalamus compared to the normal group. Protein expression of **(H)** GLP-1R, POMC and **(I)** NPY2R, AgRP in the hypothalamus. Data were shown as means ± SD (*n* = 6). Statistical analysis was performed using one-way ANOVA; ns for *p* > 0.05; *p < 0.05; **p < 0.01; ***p < 0.001; and ****p < 0.0001.

Gut hormones are essential messengers in the microbe-gut-brain axis. qPCR was performed to detect the expression of FFAR2, PYY, and GLP-1 in the gut, NPY, AgRP, POMC, and CART in the hypothalamus, and Western Blot to detect the protein expression of GLP-1R, POMC, NPY2R, and AgRP. We observed that the expression of FFAR2 was significantly lower in the *HFD* group compared to the *NCD* group. Compared with the HFD group, the relative expressions of FFAR2, PYY, and GLP-1 increased in the three TP-fed groups, and the expression of PYY and GLP-1 in the *HFD_TP_L* and *HFD_TP_H* groups was dose-dependent (Figures 5A–C). It indicated that TP caused an increase in the production of short-chain fatty acids such as acetic acid and propionic acid in the intestine of mice (Table 2), which activated the expression of its receptor FFAR2. NPY, AgRP, POMC, and CART were critical hypothalamus regulators controlling dietary intake. NPY and AgRP mainly act as food intake inhibition, whilst CART and POMC act as facilitators of feeding. Compared to the *NCD* group, the expression of NPY and AgRP was significantly increased in the *HFD* group and decreased in the *NCD_TP_H* groups, and *HFD_TP_L* and *HFD_TP_H* groups were significantly decreased compared with the *HFD* group (Figures 5D–E). CART and POMC were up-regulated in TP-fed groups (Figures 5F,G). Compared to the *HFD* group, the Western blot showed that both doses of TP caused a significant increase in the expression of GLP-1R, POMC and NPY2R and decreased the expression of AgRP (Figures 5H,I). These results suggested that TP regulated the release of intestinal hormones, which ultimately regulated the expression of regulators controlling diet in the hypothalamus to suppress obesity in mice.

### Faecal microbiota transplantation reduced obesity and improved glucolipid metabolism in mice

**Figure 6:**
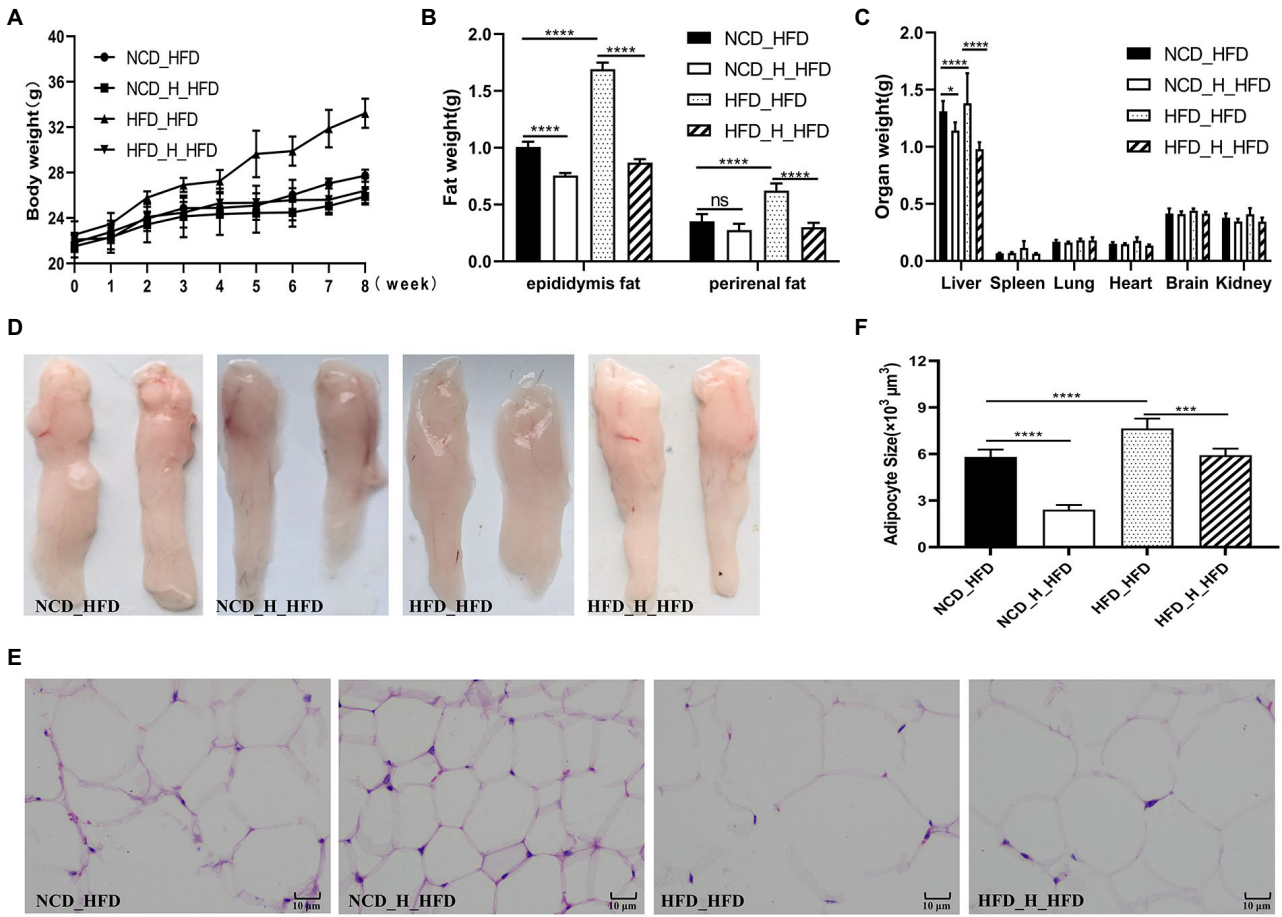
Faecal microbiota transplantation attenuated fat accumulation in HFD-fed mice. The four groups of recipient mice (fed high-fat chow) that received faecal transplants were designated as *NCD_HFD*, *NCD_H_HFD*, *HFD_HFD* and *HFD _H_HFD,* respectively. **(A)** Body weight gain. **(B)** Epididymal fat and perirenal fat weight. **(C)** Weight of different organs. **(D)** Morphological observations of the epididymis fat. **(E)** Haematoxylin and eosin staining of epididymis fat. **(F)** Epididymal adipocyte size. Data were shown as means ± SD (*n* = 6). Statistical analysis was performed using one-way ANOVA; ns for *p* > 0.05; *p < 0.05; **p < 0.01; ***p < 0.001; and ****p < 0.0001.

To investigate the effects of gut microbes on obesity in mice fed TP, we transplanted faeces from four groups (*NCD, NCD_TP_H, HFD, HFD_TP_H*) of donor mice to HFD-fed mice and then monitored the obesity-related traits as well as metabolic changes in blood glucose and blood lipids. The results showed that transplantation of faeces from *NCD*, *NCD_TP_H*, and *HFD_TP_H* mice reduced body weight (Figure 6A) and organ weight (Figure 6C), especially liver weight, and reduced fat accumulation in the recipient mice compared to the *HFD_HFD* group (Figures 6B–D). The morphological observation was that FMT inhibited the expansion of adipocytes (Figures 6E,F). Table 3 showed the changes in blood biochemical parameters in mice, similar to the results in Table 1. Compared to the *HFD_HFD* group, the other three groups had significantly lower fasting glucose, TC, TG, and LDL-C and higher levels of HDL-C, GLP-1, and GLP-2. These results suggested that the weight reduction effect of TP on HFD-fed mice may be due to the modulation of the gut microbiota.

**Table 3:** The effect of faecal microbiota transplantation on metabolic syndrome in HFD-fed mice.

Parameters	NCD_HFD	NCD_H_HFD	HFD_HFD	HFD_H_HFD
Fasting Glucose (mmol/L)	7.00 ± 0.45^c^	6.08 ± 0.39^d^	10.94 ± 0.81^a^	8.64 ± 0.75^b^
TC (mmol/L)	4.27 ± 0.04^b^	3.21 ± 0.13^c^	4.83 ± 0.07^a^	3.62 ± 0.23^b^
TG (mmol/L)	1.28 ± 0.03^b^	1.04 ± 0.02^d^	1.49 ± 0.08^a^	1.15 ± 0.05^c^
LDL-C (mmol/L)	1.67 ± 0.09^b^	0.56 ± 0.05^d^	2.55 ± 0.18^a^	1.31 ± 0.12^c^
HDL-C (mmol/L)	2.49 ± 0.16^c^	4.45 ± 0.57^a^	1.53 ± 0.13^d^	2.98 ± 0.31^b^
GLP-1 (pmol/L)	3.24 ± 0.15^c^	4.04 ± 0.13^a^	2.92 ± 0.18^d^	3.45 ± 0.16^b^
GLP-2 (pg/L)	805.57 ± 16.47^c^	971.47 ± 33.95^a^	728.15 ± 17.39^d^	844.34 ± 36.18^b^

Data were expressed as mean ± SD (*n* = 6). Significant differences were evaluated by one-way analysis of variance (ANOVA). Comparison of each parameter between the four groups, with different superscript letters indicating significant differences (*p* < 0.05).

### Faecal microbiota transplants modulated gut microbiota composition

**Figure 7:**
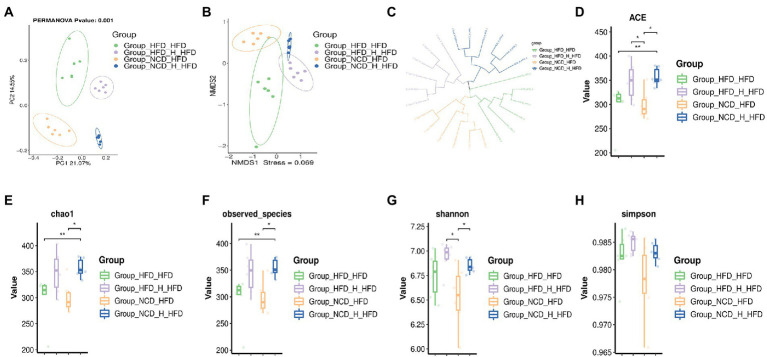
Faecal microbiota transplantation modulated the diversity of the gut microbiota. Faecal transplantation from *NCD*-, *NCD_TP_H*-, *HFD*-, and *HFD_TP_H*-fed mice was performed, and relevant microbiota analysis was conducted. Beta diversity of faecal microbiota of the four groups, including **(A)** principal components analysis (PCoA) plot, non-metric multidimensional scaling (NMDS) analysis **(B)** and hierarchical clustering **(C)**. Alpha Diversity-related boxplot analysis, including ACE **(D)**, chao 1 **(E)**, observed-species **(F)**, shannon **(G)** and simpson index **(H)**. Data were shown as means ± SD (*n* = 6). Statistical analysis was performed using one-way ANOVA; ns for *p* > 0.05; *p < 0.05; **P < 0 0.01; and ***p < 0.001.

To further explore the regulation of gut microbes by TP, we analysed the gut microbial composition of four groups of FMT mice. A beta diversity analysis included PCoA, NMDS and UPGMA sample-level clustering analysis. The differences in the composition of the intestinal microbiota of the four groups of mice were observed by PCoA (Figure 7A); 21.07 and 14.55% for PCoA1 and PCoA2, respectively, *NCD_H_HFD* and *HFD_H_HFD* groups were mainly distributed in the right quadrant, whilst the other two groups were distributed in the left quadrant, indicating significant differences in the composition of the intestinal flora of the four groups; NMDS analysis also showed similar results (Figure 7B); the results of UPGMA sample hierarchical clustering analysis showed that each of the four groups clustered into one cluster except for *HFD_HFD_6* sample (Figure 7C). In addition to this, there were significant differences in alpha-diversity between the four groups, the ACE value (Figure 7D), chao 1 value (Figure 7E) and observed_species (Figure 7F) of the *HFD_HFD* group were lower than those of the other three groups, and all three values increased after transplantation of faeces from mice fed TP. By comparing the shannon (Figure 7G) and simpson (Figure 7H) values between the four groups, we can see that the *HFD_HFD* group had lower microbial diversity than the other three groups. These results suggested that a high-fat diet altered the gut microbial structure of mice. However, transplantation of faeces from mice fed TP increased gut microbial abundance, suggesting a beneficial effect of TP on the regulation of gut microbes.

**Figure 8:**
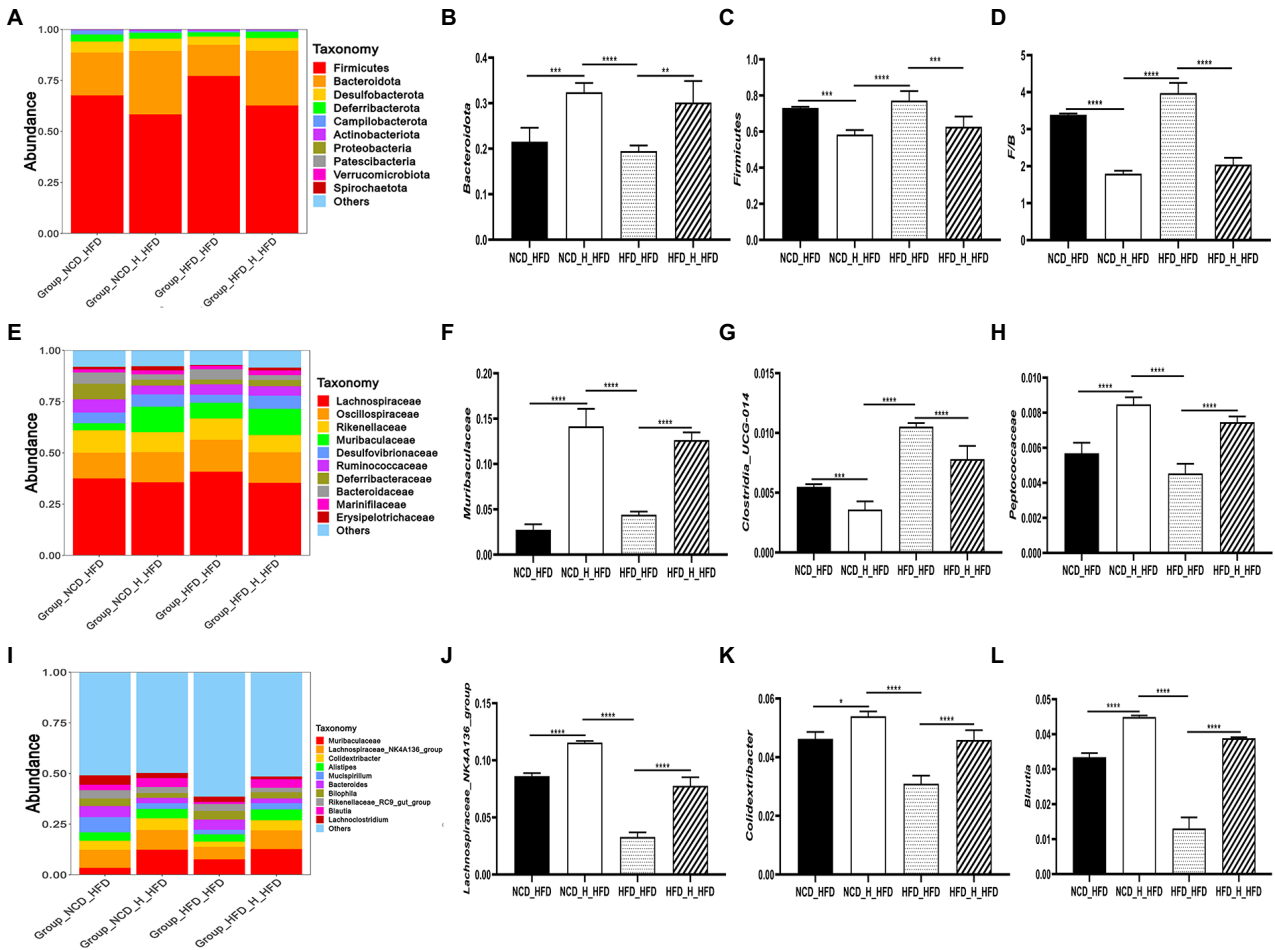
Faecal microbiota transplantation modulated the composition of the gut microbiota. **(A)** Phylum-, **(E)** family-, and **(I)** genus-level distribution of faecal microbiota. **(B,C)** relative abundance of the phyla *Bacteroidota* and *Firmicutes*; **(D)** the ratio between the relative abundance of *Firmicutes* and *Bacteroidota*. **(F-H,J-L)** relative abundance of identified differential abundant bacterial groups at family- and genus-level. Data were shown as means ± SD (*n* = 6).Statistical analysis was performed using one-way ANOVA; ns for *p* > 0.05; *p < 0.05; **p < 0.01; and ***p < 0.001.

To further investigate specific changes in the gut microbiota, we compared the microbial composition of the four groups at different taxonomic levels. At the phylum level, similar to previous results, the *HFD_HFD* group significantly altered the gut microbiota composition by increasing the *Firmicutes*/*Bacteroidota* ratio, which was reduced after transplantation of TP-fed mice faeces (Figures 8A–D). In addition, at the family level, the microbial composition of the four groups also differed significantly (Figure 8E). The relative abundance of *Actinobacteriota* was lower in the *HFD_HFD* group, whilst the relative abundance of *Proteobacteria* was higher. In the *HFD_HFD* group, the relative population abundance of *Muribaculaceae* (Figure 8F)*, Deferribacteraceae, Marinifilaceae, Bacteroidaceae, Peptostreptococcaceae, Erysipelotrichaceae, Peptococcaceae* (Figure 8G) and *Tannerellaceae* was lower, and was higher abundances of *Clostridia_UCG-014* (Figure 8H) compared to the other three groups; at the genus level, transplanted TP-fed mice faeces significantly increased the relative population abundance of *Blautia* (Figure 8L)*, Lachnospiraceae_NK4A136_group* (Figure 8J)*, Colidextribacter* (Figure 8K) and *Faecalibaculum* and decreased the relative population abundance of *Odoribacter, Tuzzerella,* and *Anaerotruncus* compared to the *HFD_HFD* group (Figure 8I). These results suggested that HFD induced changes in the gut microbiota, yet the ratio of gut microbiota was closer to that of the *NCD* group after transplanting faeces from TP-fed mice.

## Discussion

TP has been shown to regulate blood glucose and lipids (Elisashvili et al., 2002; Bach et al., 2015; Chiu et al., 2022; Zhang et al., 2023), suggesting the possible use of TP for treating obesity and diabetes. Upon proteomic analysis, the expression of 84 genes associated with obesity, insulin resistance, and diabetic complications was significantly decreased (Kim et al., 2009). Similarly, in the present study, we observed that triglycerides, cholesterol, and fasting glucose were significantly lower in mice fed TP compared to mice fed HFD. Also, our work showed that TP inhibited high-fat diet-induced weight gain, fat accumulation, and liver weight gain due to regulated gut microbiota disturbance, maintenance of the gut barrier, and increased production of SCFAs suppressing high-fat diet-induced obesity in mice through the microbe-gut-brain axis. Our results also demonstrated that TP targets the gut microbiota to suppress obesity and provided theoretical support for TP as a prebiotic agent for preventing obesity and related diseases.

Polysaccharides and dietary fibre act as energy sources for the gut microbiota and significantly impact the gut microbiota composition and the health of the host (Flint et al., 2008; Koropatkin et al., 2012; Song et al., 2021; Zhang et al., 2022). Numerous studies have shown that polysaccharides can exert a suppressive effect on obesity by modulating the gut microbiota (Shi et al., 2015; Nguyen et al., 2016; Chen Y. et al., 2018; Sang et al., 2021). We observed that TP significantly suppressed obesity in mice and modulated the gut microbiota composition of mice. *Firmicutes* and *Bacteroidetes* are the two most prevalent bacteria in the gut. They play an essential role in suppressing obesity, with many studies pointing to an increased ratio of *Firmicutes* to *Bacteroidetes* (*F/B*) as a feature of the ‘obesity microbiome’(Yan et al., 2018). In the present study, the *F/B* was significantly higher in the *HFD* group than in several other experimental groups. In contrast, the *F/B* significantly decreased after treatment with TP, which is consistent with most studies (Zhang et al., 2020; Liang et al., 2021; Li et al., 2022; Wang et al., 2022).

We also found that TP treatment of obese mice altered the relative population abundance of gut microbes. At the family level, TP treatment increased the relative population abundance of *Muribaculaceae*, *Oscillospiraceae*, *Prevotellaceae* and *Bacteroidaceae*. It decreased the relative population abundance of *Clostridia_UCG-014* and *Desulfovibrionaceae* compared to the *HFD* group. *Muribaculaceae* and *Prevotellaceae* were associated with the regulation of obesity, and the abundance of these microorganisms was lower in the obesity model (Arnoriaga-Rodríguez et al., 2020; Cao et al., 2020). The effects of *Clostridia_UCG-014* on obesity were less well studied, but a study reported that *Clostridia_UCG-014* was positively correlated with blood glucose and had a higher abundance in obesity models (Zhao et al., 2021). Similarly, a high-fat diet increased the abundance of *Desulfovibrionaceae* (Wang et al., 2020). At the genus level, the relative population abundances of *Muribaculaceae*, *Lachnospiraceae_NK4A136_group*, *Alistipes*, *Bacteroides* and *Colidextribacter* were lower in the *HFD* group, whilst *Mucispirillum*, *Bilophila*, *Blautia*, and *Clostridia_UCG-014* had higher relative population abundances.

*Colidextribacter* has been reported to produce inosine, which ameliorates acute liver injury and inflammation caused by lipopolysaccharides (Guo et al., 2021). *Bilophila*, a lipopolysaccharide-producing bacterium, exacerbates HFD-induced inflammation and causes metabolic disturbances in mice (Lu et al., 2021). Low-grade chronic mild inflammation was a feature of obesity, and pathogenic gut microbes stimulate the production and release of LPS from intestinal epithelial cells, which subsequently bind to cytokine receptors and promote the release of pro-inflammatory cytokines (Musso et al., 2012). Our study showed that TP suppressed the HFD-induced inflammatory response, mainly in the form of a reduction in serum inflammatory factors (LPS, TNF-α) and a decrease in the expression of inflammatory factor genes (IL-1β, IL-6, TNF-α) in intestinal tissues (Supplementary Figure 1). Thus, the beneficial effects of TP on obesity can be attributed to alterations in the gut microbiota and the maintenance of gut barrier integrity.

Through FMT experiments, we found that the anti-obesity effect of TP was transferred to mice fed HFD. The effect was manifested in several ways, including inhibiting body weight gain, reducing fat accumulation and expanding adipocytes. At the same time, TC, TG, and LDL-C in the serum of mice decreased significantly. Interestingly, FMT also altered the gut microbiota of obese mice. On the one hand, FMT increased the diversity and abundance of gut microorganisms in obese mice; on the other hand, the composition and structure of the gut microbiota changed significantly. At the phylum level, transplanting faeces from mice fed TP resulted in lower *F*/*B* values than the *HFD_HFD* group. At the family and genus levels, the *NCD_H_HFD* group and the *HFD_H_HFD* group had higher abundances of *Muribaculaceae, Deferribacteraceae, Marinifilaceae, Bacteroidaceae, Peptostreptococcaceae*, *Lachnospiraceae_NK4A136_group* and *Blautia*. At the same time, *Clostridia_UCG-014*, *Odoribacter* and *Bilophila* were less abundant. Comparing the gut microbiota composition of mice after TP intervention with that of mice after FMT, it can be observed that TP has a regulatory effect on gut microorganisms, whilst FMT verifies that the regulatory effect of TP on gut microorganisms is beneficial for weight loss.

*Prevotellaceae*, *Lachnospiraceae_NK4A136_group* and *Alistipes* have been reported as producers of SCFAs (Liu et al., 2016; Wang et al., 2018; Chen et al., 2022). SCFAs were another important microbial metabolite involved in the anti-obesity effect, which was metabolites of indigestible carbohydrates fermented by gut microbes in the small intestine, and played an active role in the prevention and treatment of obesity (Lu et al., 2016; Canfora et al., 2017; Weitkunat et al., 2017; Lan et al., 2022; Li et al., 2022). Our study showed that treatment of high-fat diet-induced obese mice with TP resulted in significantly higher levels of SCFAs in the faeces, particularly acetic acid, propionic acid, and butyric acid (Supplementary Figure 3).

SCFAs participate in the secretion of intestinal hormones through their receptors, such as FFAR2 and FFAR3, located in enteroendocrine cells (Brown et al., 2003; Nilsson et al., 2003). It has been found that FFAR2 is mainly responsible for stimulating the release of intestinal hormones (Tolhurst et al., 2012; Psichas et al., 2015). When SCFAs bind to the receptors, L cells located at the end of the small intestine secrete intestinal hormones such as GLP-1, GLP-2, and PYY. GLP-1 can promote insulin secretion, reduce glucagon secretion and enhance the sensitivity of the body to insulin (Namkoong et al., 2017); GLP-2 is involved in repairing and maintaining the integrity of the intestinal barrier (Odenwald and Turner, 2016); PYY can regulate intestinal motility to promote gastric emptying, produce a feeling of satiety and reduce food intake of the body (Zhou et al., 2015). Our results showed that serum levels of GLP-1 and GLP-2 were significantly higher in obese mice fed TP compared to obese model mice. In addition, the gene expression of FFAR2, GLP-1 and PYY were also significantly up-regulated in intestinal tissues, indicating that TP metabolism produced more SCFAs such as acetic acid and propionic acid, which stimulated the up-regulation of their receptor FFAR2 expression, resulting in increased secretion of two intestinal hormones, GLP-1 and PYY, by colon L cells. GLP-1R and NPY2R were receptors for GLP-1 and PYY, respectively, responsible for receiving stimuli from GLP-1 and PYY and transmitting the stimulus signals to the central system. We examined the protein expression of these two receptors in the hypothalamus and showed that feeding TP significantly up-regulated the expression of GLP-1R and NPY2R compared to the *HFD* group.

Appetite was a major driver of feeding behaviour and intake in animals and was primarily regulated by the Arcuate Nucleus (ARC), a key nucleus located in the hypothalamus. Two kinds of regulators in the ARC are closely related to appetite regulation. Neuropeptide Y (NPY) and Agouti-related Protein (AgRP) were pro-appetite regulators (Krashes et al., 2013), Pro-opiomelanocortin (POMC) and Cocaine- and Amphetamine-regulated Transcript Peptide (CART) were the anti-appetite regulators (Hill, 2010), the expression and activation of these regulators populations were directly responsible for appetite regulation and feeding behaviour. PYY and GLP1 can affect appetite and satiety by inhibiting the expression of NPY and AgRP and activating the expression of POMC and CART. In the present study, the expression of NPY and AgRP was significantly lower, and the expression of POMC and CART was significantly higher in the hypothalamus of mice fed TP compared to the HFD group; protein expression of AgRP was lower, and protein expression of POMC was higher in the hypothalamus. Unfortunately, we did not detect the protein expression of NPY and CART.

**Figure 9:**
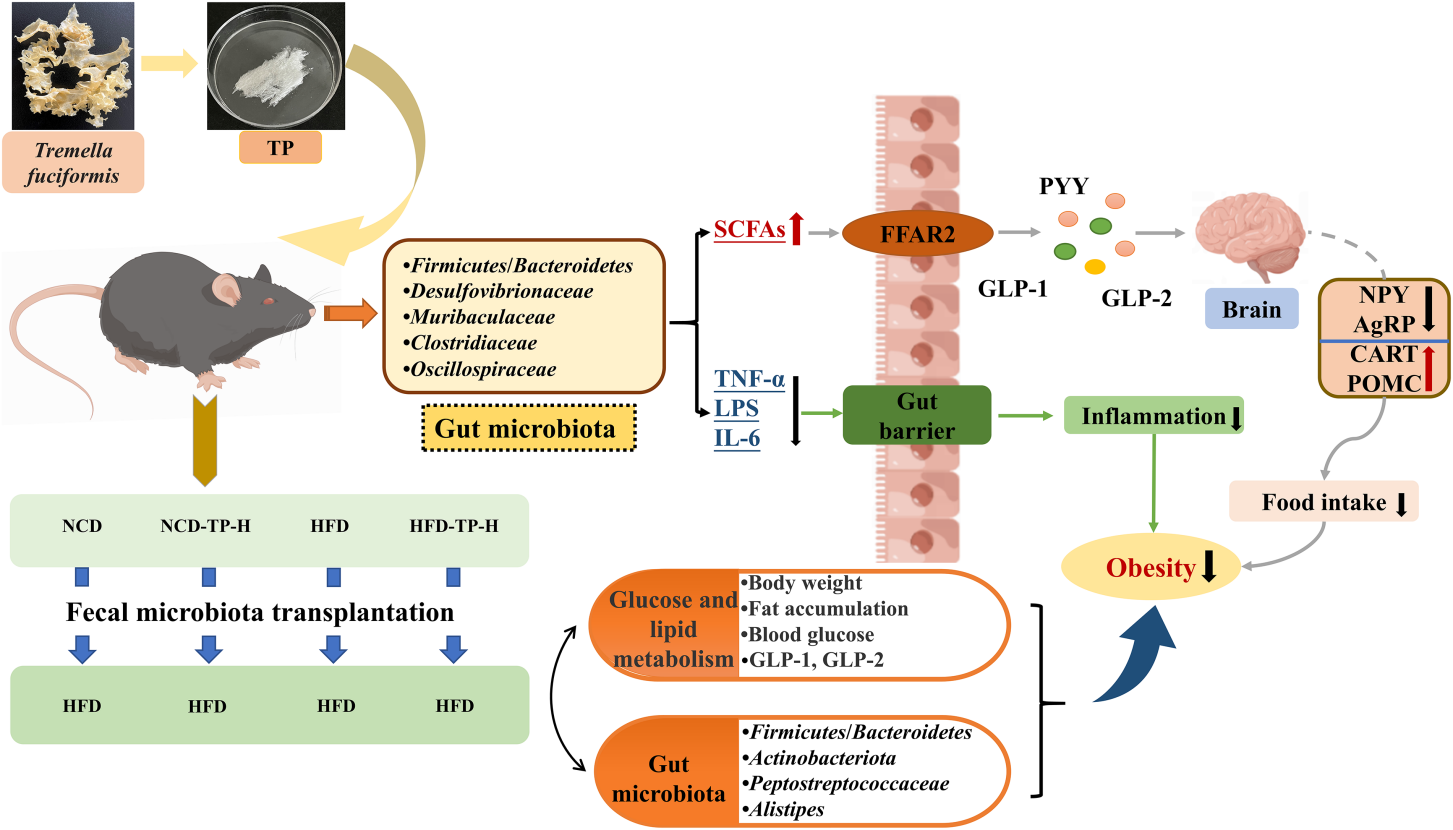
Proposed mechanisms underlying the inhibitory effects of TP on HFD-induced obesity and inflammation.

In conclusion, our study demonstrated that TP significantly reduced high-fat diet-induced weight gain, fat accumulation, inflammation, hyperglycemia, and hyperlipidemia in mice. As summarised in Figure 9, the beneficial effects of TP on obesity may be related to its modulation of gut microbiota, maintenance of gut barrier integrity, SCFAs production, and regulation of the microbe-gut-brain axis. Our findings also showed that TP could be used as a prebiotic agent to target the gut microbiota to inhibit metabolic diseases such as hyperglycemia and obesity.

## Data availability statement

The data presented in the study are deposited in the NCBI repository, accession number PRJNA901387. https://www.ncbi.nlm.nih.gov/sra/PRJNA901387.

## Ethics statement

The animal study was reviewed and approved by The Ethical Committee approved all animal experiments for the Protection of Laboratory Animals, Sichuan Industrial Institute of Antibiotics, Chengdu University (Chengdu, China; Approval Number: SIIA 20210702).

## Author contributions

GH, TC, and WL designed the study. TC and GH performed the experiments, analysed the data, and drafted the manuscript. LH, YZ, YF, SQ, XY, LL, and JY participated in partial experiments and data collection processes. GH, TC, and WL revised the manuscript. GH, TC, LH, YZ, YF, SQ, XY, LL, JY, and WL reviewed the manuscript. All authors agreed to have two corresponding authors, contributed to the article, and approved the submitted version.

## Funding

This work was supported by the National Natural Science Foundation of China (grant number: 31870655), Sichuan Science and Technology Program (grant number: 2019YFH0054 and 2020YFH0205).

## Conflict of interest

The authors declare that the research was conducted in the absence of any commercial or financial relationships that could be construed as a potential conflict of interest.

## Publisher’s note

All claims expressed in this article are solely those of the authors and do not necessarily represent those of their affiliated organizations, or those of the publisher, the editors and the reviewers. Any product that may be evaluated in this article, or claim that may be made by its manufacturer, is not guaranteed or endorsed by the publisher.

## Supplementary material

SUPPLEMENTARY FIGURE 1 TP regulated the expression of related genes. Relative expression of (A) IL-1β, (B) IL-6, (C) IL-10 and (D) TNF-α in the colon compared to the NCD group. Data were shown as means ± SD (*n* = 6). Statistical analysis was performed using one-way ANOVA; ns for *p* > 0.05, **p* < 0.05, ***p* < 0.01, ****p* < 0.001 and *****p* < 0.0001.

SUPPLEMENTARY FIGURE 2 Correlation analysis. Correlation analysis between dominant genera and obesity-related parameters, including (A) HFD vs. NCD_TP_H; (B) NCD vs. NCD_TP_H; (C) HFD vs. HFD_TP_L. (D) Heat map of the Spearman correlation between dominant genera and SCFAs. *Significance was set at *p <* 0.05.
